# Long-term results of intensity-modulated radiotherapy concomitant with chemotherapy for hypopharyngeal carcinoma aimed at laryngeal preservation

**DOI:** 10.1186/1471-2407-10-102

**Published:** 2010-03-18

**Authors:** Wen-Shan Liu, Chung-Han Hsin, Ying-Hsiang Chou, Jung-Tung Liu, Ming-Fang Wu, Szu-Wen Tseng, Jong-Kang Lee, Hsien-Chun Tseng, Tzu-Hwei Wang, Mao-Chang Su, Huei Lee

**Affiliations:** 1 Departments of Radiation Oncology, Chung Shan Medical University Hospital, No 110, Sec 1, Chien-Kuo N Rd, Taichung 402, Taiwan; 2 Otolaryngology, Chung Shan Medical University Hospital, No 110, Sec 1, Chien-Kuo N Rd, Taichung 402, Taiwan; 3 Neurosurgery, Chung Shan Medical University Hospital, No 110, Sec 1, Chien-Kuo N Rd., Taichung 402, Taiwan; 4 Internal Medicine Division of Medical Oncology, Chung Shan Medical University Hospital, No 110, Sec 1, Chien-Kuo N Rd, Taichung 402, Taiwan; 5 Nuclear Medicine, Chung Shan Medical University Hospital, No 110, Sec 1, Chien-Kuo N Rd, Taichung 402, Taiwan; 6 Department of Radiation Oncology, Taichung Tzu Chi General Hospital, No 66, Sec 1, Fongsing Rd, Tanzih Township, Taichung 427, Taiwan; 7 School of Medicine, Chung Shan Medical University, No 110, Sec 1, Chien-Kuo N Rd, Taichung 402, Taiwan

## Abstract

**Background:**

The objective of this retrospective study is to investigate laryngeal preservation and long-term treatment results in hypopharyngeal carcinoma treated with intensity-modulated radiotherapy (IMRT) combined with chemotherapy.

**Methods:**

Twenty-seven patients with hypopharyngeal carcinoma (stage II-IV) were enrolled and underwent concurrent chemoradiotherapy. The chemotherapy regimens were monthly cisplatin and 5-fluorouracil for six patients and weekly cisplatin for 19 patients. All patients were treated with IMRT with simultaneous integrated boost technique. Acute and late toxicities were recorded based on CTCAE 3.0 (Common Terminology Criteria for Adverse Events).

**Results:**

The median follow-up time for survivors was 53.0 months (range 36-82 months). The initial complete response rate was 85.2%, with a laryngeal preservation rate of 63.0%. The 5-year functional laryngeal, local-regional control, disease-free and overall survival rates were 59.7%, 63.3%, 51.0% and 34.8%, respectively. The most common greater than or equal to grade 3 acute and late effects were dysphagia (63.0%, 17 of 27 patients) and laryngeal stricture (18.5%, 5 of 27 patients), respectively. Patients belonging to the high risk group showed significantly higher risk of tracheostomy compared to the low risk group (p = 0.014).

**Conclusions:**

After long-term follow-up, our results confirmed that patients with hypopharyngeal carcinoma treated with IMRT concurrent with platinum-based chemotherapy attain high functional laryngeal and local-regional control survival rates. However, the late effect of laryngeal stricture remains a problem, particularly for high risk group patients.

## Background

Patients with resectable, locally advanced laryngeal and hypopharyngeal carcinoma have historically been treated with surgery and adjuvant radiotherapy [1-3]. However, their quality of life is greatly impaired because the entire larynx is sacrificed. After the finding by VA Laryngeal Cancer Study Group [4] that induction chemotherapy followed by definitive radiotherapy leads to a larynx preservation rate of 68%, this method has been considered as an organ preservation treatment for laryngeal carcinoma [5-8]. The European Organization for Research and Treatment of Cancer (EORTC) conducted a phase III study for hypopharyngeal carcinoma aimed at laryngeal preservation (EORTC 24891) showing that treatment with induction chemotherapy and radiotherapy yields comparable local and overall survivals to treatment with surgery and adjuvant radiotherapy [5]. The Southwest Oncology Group designed a phase II trial for patients with cancer of the base of the tongue or hypopharynx aimed at organ preservation [7]. Induction chemotherapy was carried out first then selected patients with more than 50% response were given concurrent chemo-radiotherapy. Their results showed that 75% of patients did not require surgery for the primary tumor. With the success of induction chemotherapy and radiotherapy for laryngeal preservation while maintaining local control and survival for laryngeal or hypopharyngeal carcinoma, other studies on the efficacy of concurrent chemotherapy have been conducted, such as the Radiation Therapy Oncology Group (RTOG) study 91-11 [9]. That study evaluated the laryngeal preservation rates at 2 years, and the preservation rate in the concurrent chemoradiotherapy group (88%) was significantly higher than that in the induction chemotherapy followed by radiotherapy group (75%, p = 0.005) and the radiotherapy alone group (70%, p < 0.001) [9]. However, the major concern with the concurrent chemoradiotherapy approach is profound acute side-effects [9,10]. Hence, the EORTC (24954) designed a protocol of alternating chemotherapy and radiotherapy to compare sequential treatments [11].

More recently, intensity-modulated radiotherapy (IMRT) has been adopted for reduction of treatment related side-effects in head and neck cancer patients [12-17]. Dosimetry studies have confirmed that IMRT method can deliver more precise dose distribution around the target(s) and maintain lower doses to critical nearby organs [18,19]. Clinical studies of nasopharyngeal cancer patients treated with IMRT confirm that this technique reduces the degree of xerostomia while maintaining and even increasing local control of this disease [13,20]. Although there is widespread application of IMRT for head and neck tumor, only a few studies have reported the effectiveness of laryngeal preservation and treatment outcomes of hypopharyngeal cancer treated with this new technology [21,22]. Moreover, these studies had a relatively short follow-up period. For example, the median follow-up time in Lee's study was 26 months [22]. To properly evaluate the role of IMRT in this disease, it is very important to understand the long-term results, including both the laryngeal preservation survival and late complications for hypopharyngeal carcinoma. Therefore, the primary purpose of this retrospective study is to investigate the long-term effectiveness of laryngeal preservation and treatment outcomes in hypopharyngeal carcinomas treated with IMRT and concomitant chemotherapy. The secondary purpose is to evaluate the severity of late toxicities correlated with this treatment.

## Methods

### Patients and staging

Between May 2001 and February 2005, a single cohort of 27 consecutive men (mean age 60.7 years; age range 42 to 85 years) with previously untreated stage II-IV squamous cell carcinoma of hypopharynx were included in this study. All patients had histological proved disease and had refused surgical management or had initially unresectable disease. None had a previous or synchronous malignancy. Their disease was staged according to the 2002 classification of the American Joint Committee on Cancer. The distribution of T and N status are listed in Table 1. Five patients had stage II disease, four patients had stage III disease, sixteen patients had stage IVa disease, and two patients had stage IVb disease.

**Table 1:** Tumor and lymph node classifications of study participants

	Node Stage			
	
Tumor Stage	N0	N1	N2	N3
T2 (n = 8)	5	1	1	1
T3 (n = 6)	2	1	3	0
T4 (n = 13)	1	2	8	2
**Total (n = 27)**	**8**	**4**	**12**	**3**

*Tumor and nodes were staged according to the 2002 classification of the American Joint Committee on Cancer Staging.

Medical histories were reviewed, and all patients underwent physical examination, laryngoscopic assessment, analysis of complete blood count, blood biochemistry testing, chest radiography, computerized tomography or magnetic resonance imaging (MRI), and dental evaluation. Informed consent was obtained from all patients before receiving treatment, and this study was approved by the Institutional Review Board of Chung Shan Medical University Hospital.

### Radiotherapy

All patients were treated in the supine position with the use of immobilization masks and rigid pillows to support the neck (MT-201-D, MEDTEC Inc.). With computerized tomographic simulator (GE HiSpeed Fx, GE Inc.) series images of 3-mm per section were acquired. The definitions of gross target volume (GTV), clinical target volume (CTV), and planning target volume (PTV) followed those of the International Commission on Radiation Units and Measurements, ICRU report 62 [23]. The PTV-1 and PTV-2 were extended from GTV and CTV three-dimensionally with 5 mm and 3 mm margins, respectively. All patients received two stages of radiotherapy; the first stage was composed of PTV-1 and PTV-2 and the second stage only covered PTV-1 for boosting the gross tumor. This treatment plan was considered acceptable if more than 99% volume of PTV-1 and 98% volume of PTV-2 could be covered by the prescribed dose. In the first stage of treatment, PTV-1 was treated with a dose of 63.6 Gy in 30 fractions for T2-T3 lesions and 67.8 Gy in 32 fractions for T4 lesions. We used the principle of simultaneous integrated boost (SIB) in the treatment plan [18]. The fraction sizes for PTV-1 and PTV-2 were 2.12 Gy and 1.75~1.8 Gy, respectively. In the second stage of treatment, PTV-1 was planned for another 9.0 Gy in five fractions. With the above two stages, the planned total doses to PTV-1 for T2-3 and T4 cases were 72.6 Gy in 35 fractions and 76.8 Gy in 37 fractions, respectively. Hence, the total doses to PTV-2 were 54 Gy and 56 Gy for T2-3 and T4 diseases, respectively. All treatment plans were calculated by Helio treatment planning system (Varian Oncology Systems, Palo Alto, CA). According to a study by Wu [12], the fine-tuning of beam angles is not important in IMRT, in contrast to standard radiotherapy, as long as a sufficient number of beam angles are used. Therefore, we defined seven gantry angles arbitrarily. They are 20°, 70°, 125°, 175°, 220°, 275°, and 325°. Twenty segments of each treatment field were set with step-and-shot method. Then, the intensity distributions within the treatment fields were optimized to gratify constraints of different normal tissues and targets like mentioned above. Treatment was delivered using a dynamic multileaf collimator system (Varian Oncology Systems, Palo Alto, CA).

Elective irradiation of regional lymph nodes was designed using the same principles in all cases. These lymphatic areas, including in the CTV, were bilateral levels of Ib, II, III, IV, and V, plus central area of VI, as defined by the American Joint Committee on Cancer. The bilateral retropharyngeal lymph nodes were also included in the CTV volume. Bilateral supraclavicular treatment field was defined as PTV-3 and arranged by an anterior-posterior portal. When treating this supraclavicular treatment field, we did not use a central block to protect the spinal cord until the dose accumulated to 43.2 Gy in 24 fractions. Then, this field was further irradiated to 48.6 to 52.2 Gy in 27 to 29 fractions with central shielding. Same iso-center and positioning setup was used for both supraclavicular (PTV-3) and IMRT fields (PTV-1 and PTV-2) in order to minimize junction errors between them. The constraints of nearby organs were spinal cord maximum lower than 45 Gy, ipsilateral parotid gland mean dose lower than 30 Gy, and contralateral parotid gland lower than 26 Gy. If the tumor extended near to or invaded the vocal cords, no specific protection to this region was planned. Otherwise, we tried to restrict the dose to opposite vocal cord to below 60 Gy.

### Chemotherapy

All patients received concomitant chemoradiotherapy. Early in the study, six patients received two courses of chemotherapy during radiotherapy. Their regimen was cisplatin 60 mg/m^2^ on days 1 and 29, and 5-fluorouracil 600 mg/m^2^ on days 1-4 and 29-32. This regimen resulted in profound pharyngitis and esophagitis, thus the remaining 21 patients received cisplatin 30 mg/m^2^/week for 7 weeks.

### Treatment evaluation and follow-up

During treatment, patients were examined weekly. After treatment was completed, patients were evaluated every month for the first year, every 3 months for the second and third years, and every 6 months until last follow-up. Follow-up time was defined as the start of radiotherapy to May 2008. The treatment response of the primary tumor was evaluated by means of both fiberscope laryngoscope and MRI examination two to three months after completion of radiotherapy. Then, fiberscope laryngoscope was performed every three months for the first two years, and every 6 months until last follow-up. MRI examination was done annually until last follow-up. Seven patients were evaluated by FDG PET scan about five to six months after treatment. The definition of residual or recurrent disease was judged by both fiberscope laryngoscope and MRI examination. The complete response was defined by clinical assessment and tissue biopsy was carried out if there was any uncertainty. The acute and late adverse effects were graded according to the criteria of CTCAE 3.0 [24].

### Statistical analysis

Functional laryngeal, local-regional progression-free, disease-free, and overall survival rates were calculated using the Kaplan-Meier method. To evaluate the feasibility and toxicity of IMRT treatment modality we evaluated the risk factors for grade-3 late toxicity. The Fisher exact test was used to evaluate the correlation between toxicity scales (including tracheostomy) and potential risk factors such as T-stage (T2-3 vs. T4), GTV dose (less than 76 Gy vs. more than 76 Gy), gross tumor volume (less than 37 ml vs. more than 37 ml), primary tumor location (pyriform sinus vs. other sites) and chemotherapy regimens (cisplatin alone vs. cisplatin plus 5-fluorouracil), respectively. To evaluate any potential risk group that may correlate of receiving tracheostomy during or after this treatment, we separated our patients into two risk groups based on below definitions. We defined the high risk group as possessing two to three of the following factors: GTV dose more than 76 Gy, gross tumor more than 37 ml and primary tumor location other than pyriform sinus. Those without or just one of the above factors were defined as low risk group. The Fisher exact test was used to evaluate the correlation between tracheostomy and the high risk group. The Log Rank test was used to evaluate the functional laryngeal survival difference between high and low risk groups. Statistical significance was set at p < 0.05.

## Results

### Doses to targets and critical tissues

The mean total doses to PTV-1, PTV-2 and PTV-3 were 76.2 Gy, 60.6 Gy and 51.3 Gy, respectively. Table 2 presents the mean doses to targets and important surrounding normal organs. All doses to targets and critical organs were the summations of all treatment plans including 3D CRT and IMRT.

**Table 2:** Doses to targets and critical organs in larynx-preservation treatment of hypopharyngeal carcinoma

	Mean (Gy)	Range (Gy)	Dose (Gy)/no. **
PTV-1*	76.2	70.1--82.7	2.12/33-37
PTV-2*	60.6	54.7--67.2	1.75-1.8/34-37
PTV-3*	51.3	48.6-57.6	1.8/27-32
Spinal Cord§	49.1	39.3--60.6	
Spinal Cord†	44.5	36.2--50.0	
Parotid Gland, ipsilateral	34.1	23.3--46.6	
Parotid Gland, contralateral	30.5	18.7--44.7	

* PTV-1, planning target volume of gross tumor; PTV-2, planning target volume of clinical target volume; PTV-3, planning target volume of bilateral supraclavicular fossa.

§Maximal dose to the spinal cord.

†Five percent volume dose to the spinal cord.

** Daily dose and fraction numbers in IMRT treatment.

### Follow-up time

The median follow-up time was 36.0 months (range 2 to 82 months) for all patients, and 53.0 months (range 36 to 82 months) for survivors.

### Treatment outcomes and laryngeal preservation

**Figure 1:**
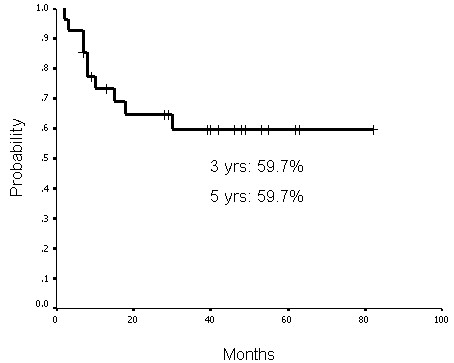
**Functional larynx survival rates at 3 years and 5 years were 59.7% and 59.7%, respectively**.

**Figure 2:**
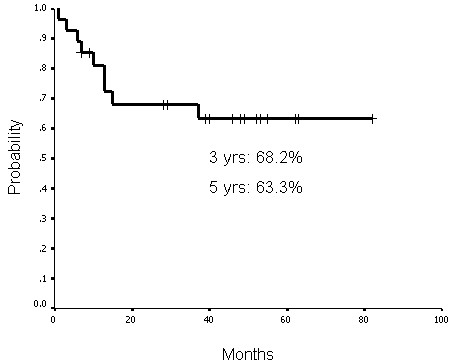
**Local-regional progression-free survival rates at 3 years and 5 years were 68.2% and 63.3%, respectively**.

**Figure 3:**
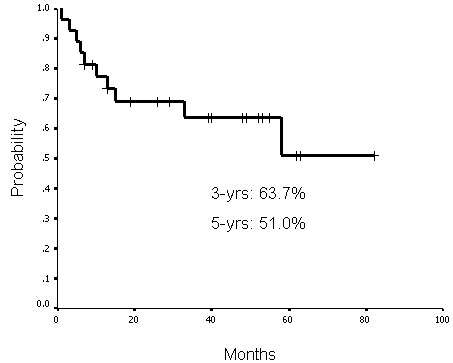
**Disease-free survival rates at 3 years and 5 years were 63.7% and 51.0%, respectively**.

**Figure 4:**
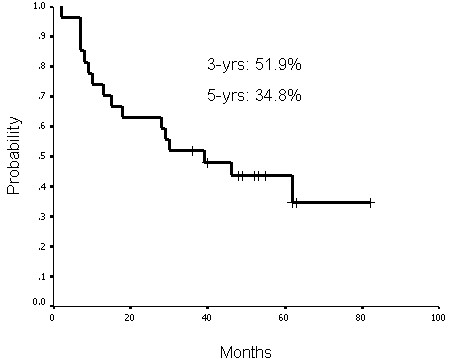
**Overall survival rates at 3 years and 5 years were 51.9% and 34.8%, respectively**.

The functional laryngeal preservation rate was 63.0% (17 of 27 patients) up to the last follow-up (May 2008). The complete response rate after treatment was 85.2% (23 of 27 patients). Three patients had persistent disease at the primary sites and two patients at the neck lymph nodes, one of them had persistent disease at both sites. Up to the last follow-up, four patients had primary recurrence. Of those patients, three had disease at the primary site and one had disease at both the primary site and neck lymph nodes. The functional laryngeal, local-regional progression-free, disease-free, and actuarial overall survival rates at 3 years were 59.7%, 68.2%, 63.7%, and 51.9%, respectively. The functional laryngeal, local-regional progression-free, disease-free, and actuarial overall survival rates at 5 years were 59.7%, 63.3%, 51.0%, and 34.8%, respectively (Figures 1, 2, 3 and 4).

Two patients received salvage surgery for local-regional failure: one due to persistent disease (primary site and lymph neck nodes) and the other due to primary recurrence 13 months after radiotherapy. At the last follow-up, these two patients were still alive without evidence of recurrence. Regarding distant metastases, three patients had lung metastases 13 to 33 months after treatment and three patients had bone metastases 5 to 58 months after treatment. Two patients developed secondary malignancies, including gastric and colon cancer 10 and 17 months after radiotherapy, respectively. One patient received radical surgery. At the last follow-up, one of these two patients was alive without evidence of disease while the other had died of gastric cancer.

### Adverse effects

**Figure 5:**
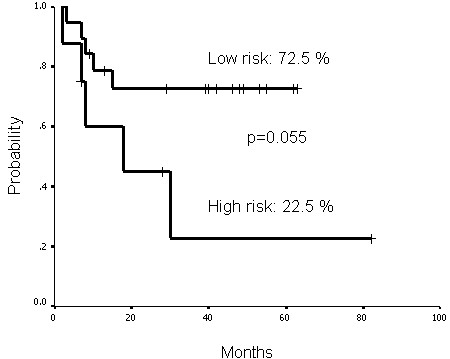
**Functional larynx survival rates of the high and low risk groups at 5 years were 22.5% and 72.5%, respectively**. The high risk group was defined as those possessing two or more factors including GTV dose of more than 76 Gy, gross tumor more than 37 ml and/or primary tumor location other than pyriform sinus. The low risk group was defined as those with one or none of the above factors.

Table 3 lists the sites and grades of the acute and late effects. The most common site of greater than or equal to grade 3 acute side-effects was the dysphagia (63.0%, 17 of 27 patients). The most common site of greater than or equal to grade 3 late effects was the larynx (stricture) (18.5%, 5 of 27). There were no significant correlations between greater than or equal to grade 3 late laryngeal toxicity and T4 stage (p = 0.62), GTV dose greater than 76 Gy (p = 0.27), volume of gross tumor greater than 37 ml (p = 0.19) or location of the primary tumor (p = 0.61). When we evaluated the correlation between the ratio of this late effect and the risk groups, there was significant difference (p = 0.014) between the high (5 of 8 patients) and low risk groups (2 of 16 patients) on Fisher exact test. However, there was no significant difference in functional laryngeal survival between these two groups (p = 0.055, figure 5). The ratios of greater than or equal to grade 3 acute toxicity of the pharynx for the monthly regimen of cisplatin plus 5-fluorouracil and the weekly regimen of cisplatin were 83.3% (5 of 6 patients) and 47.6% (10 of 21 patients), respectively. However, the difference was not statistically significant (p = 0.277). Sixteen patients (59.3%) required nasogastric-tube feeding during the treatment while seven patients (25.9%) required a tracheostomy due to stridor. However, only two of them (2/7) could reverse from tracheostomy to normal breathing at three and six months, respectively.

One patient (3.7%) died of pharyngeal late effects 10 months after treatment due to aspiration pneumonia caused by severe pharyngeal stricture and poor swallowing function. This patient had T2N3M0 disease and had received chemotherapeutic regimen of weekly cisplatin. The radiation doses to primary tumor and neck lymph nodes were 76.2 and 77.5 Gy, respectively.

**Table 3:** Acute and late adverse effects of larynx-preservation treatment of hypopharyngeal carcinoma

	Grade			
	
Site and Effect	0	1	2	≥3†
Skin				
Acute, dermatitis	3	9	11	3
Late, telangiectasia	14	6	4	1
Late, fibrosis	9	11	4	1
Mucositis				
Acute, oral cavity	0	4	13	9
Pharynx/esophagus				
Acute, dysphagia	0	1	9	17
Late, stricture	2	15	5	1
Larynx				
Acute, edema	0	4	14	8
Late, stenosis	3	13	4	5
Xerostomia				
Acute	0	11	13	2
Late	1	12	11	1
Myelitis				
Late	24	3	0	0

* The acute and late adverse effects were graded according to the scoring criteria of the CTCAE 3.0 [24].

† All effects were grade 3, with the exception of the grade 5 late effects in the pharynx/esophagus.

Data are given as number of incidences.

## Discussion

Patients with hypopharyngeal carcinoma usually have functional and cosmetic sequelae after surgery. Therefore, any attempt to preserve the larynx while achieving the treatment outcomes equal to surgical results is warranted. Most studies of laryngeal preservation have included hypopharyngeal carcinoma, as well as carcinomas of the tongue base, oropharynx, and larynx [6,7,25,26]. To our knowledge, with median follow-up time of 53.0 months (range 36 to 82 months); this is the longest follow-up of the clinical experience of IMRT concurrent with chemotherapy for hypopharyngeal cancer so far. The importance of long-term follow-up is to evaluate the late effect and laryngeal preservation rate of IMRT treatment.

The proportion of severe acute toxicity with concomitant chemoradiotherapy is expected to be higher than that of radiotherapy alone [9,10,27]. In this study, the most common greater than or equal to grade 3 acute side effects was dysphagia (17 of 27 patients, 63.0%). In Lee's report, all patients experienced grade 2 pharyngitis [22], and all patients received feeding tubes via percutaneous endoscopic gastrostomy (PEG) (prophylactic in 30 and during treatment in one). Thus, it is difficult to evaluate the true acute dysphagia grading from their series. As for the late effect causing more than grade 3 dysphagia, De Arruda et al. reported their experience of IMRT treatment for oropharyngeal cancer [28]. They performed PEG prior to radiotherapy for 42 of 50 patients (84%) and during radiotherapy for 6 (12%). Eisbruch et al. demonstrated that the sparing of pharyngeal constrictor muscle, supraglottic larynx and glottic larynx can reduce the complication of late dysphagia for those receiving chemoradiotherapy with IMRT technique [29]. From our study, the mean dose to the above-mentioned three anatomical structures was 60.6 Gy (PTV-2) unless gross tumor presented within these areas. The incidence of grade 3 acute dysphagia was relatively high, 16 patients needed NG tube and one patient refused NG tube or PEG insertion. Fortunately, this acute adverse effect was manageable, and fifteen of our patients were able to have the NG tube removed one to six months after completion of treatment. Using IMRT for protection of normal tissue from extensive radiation damage, there was only one long-term survivor (3.7%) who suffered from grade 3 late dysphagia caused by pharyngo-esophageal stricture. However, the incidence of late dysphagia may be underestimated due to some patients died early from disease, and thus there may not have been enough time to develop late effect. With careful monitoring and management of all acute treatment side effects, no patient died of acute reaction or complication during this multi-modality aggressive treatment.

Few studies have addressed acute or late effects of stridor caused by laryngeal edema or stricture, respectively. Recently, Nangia et al. reported results of 83 head and neck cancers treated by IMRT method [30]. In their series, hypopharyngeal cancer was present in 13 and laryngeal cancer in 35 patients. Concerning tracheostomy, there were five patients in whom this procedure was performed before irradiation and eight patients in who it was performed after irradiation. Among these 13 patients who received tracheostomy, there were 5 patients with closure after the completion of treatment. However, they did not evaluate the risk factors for predicting this adverse effect. In our study, seven patients needed tracheostomy during or within 3 months after CCRT, and only two of them could closure thereafter. It is imperative to perform tracheostomy surgery when there is grade 3 acute/late laryngeal strictures. Due to low reversibility of this side-effect, we attempted to evaluate the predictive value of risk factors that may correlate to this late effect. These potential risk factors included T4-stage (p = 0.62), GTV dose greater than 76 Gy (p = 0.274), volume of gross tumor greater than 37 ml (p = 0.19) and location of primary tumor other than the pyriform sinus (p = 0.61). However, we did not yield any meaningful results between these risk factors and the ratio of late laryngeal stricture. Interestingly, if we divided our patients into high and low risk groups, significant differences (p = 0.014) were found for the ratio of tracheostomy. The definition of the high risk group was those possessing two to three of the following factors: GTV dose more than 76 Gy, gross tumor more than 37 ml and primary tumor location other than pyriform sinus. Those without or only one of the above risk factors were defined as low risk group. When we evaluated the functional laryngeal survival between these two groups, there was no significant difference (p = 0.055, Figure 5). However, the trend of better functional laryngeal survival for low risk group was clearly demonstrated.

As this is only a retrospective study, any attempt to compare our survival data with randomized trials is difficult or even impossible. We list recently studies that focused on the organ preservation for hypopharyngeal cancer in Table 4. Only a few studies have focused on laryngeal preservation by IMRT for hypopharyngeal carcinoma [21,22]. The earliest clinical experience of IMRT for head and neck tumor is for nasopharyngeal carcinoma [13]. Lee et al. conducted one of the first clinical studies to focus on laryngeal preservation with IMRT technique for laryngeal (20 cases) and hypopharyngeal (11 cases) carcinoma [22]. They found that the 2-year local progression free survival rate is 86% with acceptable acute side-effects. This study shows that SIB-IMRT and concomitant chemotherapy is highly effective in the treatment of hypopharyngeal carcinoma. After a median follow-up time of 53.0 months, the goal of functional laryngeal preservation was achieved in 17 of 27 patients (63.0%). Local-regional control survival rates at 3 years and 5 years were 68.2% and 63.3%, respectively. However, the 3- and 5-year disease-free survival rates (63.7% and 51.0%) and overall survival rates (51.9% and 34.8%) are not compatible with the survival rate of local-regional control. Our long-term follow-up results indicate that even if local-regional control is stable after three years, distant metastatic events and secondary primary malignancy can impair overall survival over time. Therefore, there exists the great challenge of reducing the incidences of distant metastases and secondary malignant disease among these patients.

**Table 4:** Results of treatment in hypopharyngeal cancer with organ preservation approach

Author	Methods	No.	LCS (yr)	DFS (yr)	OS (yr)	LPS (yr)
Lefebvre[5]	Surgery	94		31% (3)	43% (3)	-
	IC + RT	100		43% (3)	57% (3)	42% (3)
Zelefsky[8]	IC + RT	26	30% (5)		15% (5)	52% (5)
	Surgery	30	42% (5)		22% (5)	
Altundag[6]	IC + RT	45 total (5 hypo.)	50.9% (2)			63.3% (2)
Urba[7]	IC + RT	59 total (22 hypo.)			64% (3)	52% (3)
Lee [22]	CCRT	31 (11 hypo.)	94% (2)	86% (2)	63% (2)	89% (2)
Lefebvre[11]	IC + RT	224 (116 hypo.)	49.7% (3)		48.5% (5)	39.5% (5)
	alternating	226 (115 hypo.)	50.6% (3)		51.9% (5)	45.4% (5)
This study	CCRT	27	63.0% (5)	51% (5)	34.8% (5)	59.2% (5)

LCS: local control survival, DFS: disease-free survival, OS: overall survival, LPS: laryngeal preservation survival, IC: induction chemotherapy, RT: radiotherapy, hypo.: hypopharynx, HF-ACC-RCT: hyperfractionated accelerated radiochemotherapy, HF-ACC-RT: hyperfractionated accelerated radiotherapy, CCRT: concurrent chemoradiotheapy.

## Conclusions

The main concerns of this new treatment are feasibility and toxicity. Our experience found that the most common grade 3 acute adverse effect is dysphagia. Fortunately, this could be managed with medication and nutritional support, e.g. insertion of NG tube. The most common grade 3 late toxicity was laryngeal stricture. This toxicity impairs both the functional laryngeal preservation rate and survival. It is imperative to perform tracheostomy surgery when there is grade 3 acute or late laryngeal stricture. To prevent this late effect, our study found that patients in the high risk group have significantly higher risk of receiving tracheostomy. As for tumor control and survival after this aggressive CCRT protocol, our experience yielded high functional laryngeal and local-regional control survival at 3- and 5-years. However, with long-term follow-up, the disease-free survival and overall survival rates declined gradually despite high local-regional control survival. These findings encourage further investigation of concurrent chemo-radiotherapy with SIB-IMRT technique for hypopharyngeal carcinoma both for laryngeal preservation and disease control.

## Competing interests

The authors declare that they have no competing interests.

## Authors' contributions

WL and JL participated in the study conception, design, data acquisition, interpretation of data and drafting of the manuscript. CH and HL participated in the study design, data acquisition, and drafting of the manuscript. YC and TW participated in data acquisition and analysis. MW and ST participated in the study conception, design and drafting of the manuscript. JL and HT participated in data analysis. MS participated in the interpretation of data and drafting of the manuscript. All authors have read and approved the final manuscript.

## Pre-publication history

The pre-publication history for this paper can be accessed here:

http://www.biomedcentral.com/1471-2407/10/102/prepub
